# PPARγ-Independent Side Effects of Thiazolidinediones on Mitochondrial Redox State in Rat Isolated Hearts

**DOI:** 10.3390/cells9010252

**Published:** 2020-01-20

**Authors:** Matthias L. Riess, Reem Elorbany, Dorothee Weihrauch, David F. Stowe, Amadou K.S. Camara

**Affiliations:** 1 Anesthesiology, TVHS VA Medical Center, Nashville, TN 37212, USA; 2 Department of Anesthesiology, Vanderbilt University Medical Center, Nashville, TN 37232, USA; 3 Department of Pharmacology, Vanderbilt University, Nashville, TN 37232, USA; 4 Interdisciplinary Scientist Training Program, University of Chicago, Chicago, IL 60637, USA; reemelorbany@uchicago.edu; 5 Department of Anesthesiology, Medical College of Wisconsin, Milwaukee, WI 53226, USA; dorothee@mcw.edu (D.W.); dfstowe@mcw.edu (D.F.S.);; 6 Department of Physiology, Medical College of Wisconsin, Milwaukee, WI 53226, USA; 7 Department of Biomedical Engineering, Medical College of Wisconsin, Milwaukee, WI 53226, USA; 8 Clement J. Zablocki VA Medical Center, Milwaukee, WI 53295, USA

**Keywords:** GW9662, ischemia reperfusion injury, Langendorff, myocardial, pioglitazone, redox state, rosiglitazone, TZD, uncoupling

## Abstract

The effect of anti-diabetic thiazolidinediones (TZDs) on contributing to heart failure and cardiac ischemia/reperfusion (IR) injury is controversial. In this study we investigated the effect of select TZDs on myocardial and mitochondrial function in Brown Norway rat isolated hearts. In a first set of experiments, the TZD rosiglitazone was given acutely before global myocardial IR, and pre- and post-IR function and infarct size were assessed. In a second set of experiments, different concentrations of rosiglitazone and pioglitazone were administered in the presence or absence of the specific PPARγ antagonist GW9662, and their effects on the mitochondrial redox state were measured by online NADH and FAD autofluorescence. The administration of rosiglitazone did not significantly affect myocardial function except for transiently increasing coronary flow, but it increased IR injury compared to the control hearts. Both TZDs resulted in dose-dependent, reversible increases in mitochondrial oxidation which was not attenuated by GW9662. Taken together, these data suggest that TZDs cause excessive mitochondrial uncoupling by a PPARγ-independent mechanism. Acute rosiglitazone administration before IR was associated with enhanced cardiac injury. If translated clinically, susceptible patients on PPARγ agonists may experience enhanced myocardial IR injury by mitochondrial dysfunction.

## 1. Introduction

Thiazolidinediones (TZDs) are a class of anti-diabetic drugs that sensitize fat cells to insulin [1] through activation of the peroxisome proliferator-activated receptor-gamma (PPARγ). PPARγ activation has been postulated to activate endothelial nitric oxide synthase, which plays a key role in cardioprotection [2,3]. Therefore, TZDs have cardioprotective effects in ischemia/reperfusion (IR) injury, as reported both in in- and ex-vivo models [4,5,6,7,8,9,10]. Moreover, since the PPARγ antagonist GW9662 abolishes both endogenous [11] and exogenous [11,12] cardioprotection against IR injury, the notion that TZDs could be beneficial indirectly through controlling diabetes and through direct cardioprotection against IR injury appears attractive.

However, reports about the deleterious cardiovascular side effects of one of the TZDs, rosiglitazone, first in animals [13,14,15,16,17] and later in humans [18,19] have dampened these hopes. As a result rosiglitazone was taken off the market in Europe and had been put under sales restriction in the USA for several years [20,21]. Pioglitazone, another popular TZD, has a better cardiovascular safety profile [22], but was subsequently taken off the market in several countries after reports of an increased incidence of bladder cancer [23,24,25]. Although rosiglitazone was subsequently not found to be associated with increased ischemic events [26] and the sales restrictions were lifted in the USA, both drugs remain contra-indicated in patients with heart failure [27,28].

One potential mechanism of this process may involve mitochondria, which play a key role in cell signaling and cell death, and can attenuate or aggravate IR injury [29]. The goal of our study was to investigate if TZDs affect the mitochondrial redox state in rat isolated hearts.

## 2. Material and Methods

Our isolated heart model has been described in detail [30,31,32,33]. All drugs were purchased from Sigma (St. Louis, MO, USA) unless otherwise indicated. Rosiglitazone, pioglitazone, and GW 9662 were dissolved in dimethyl sulfoxide (DMSO) and 1000-fold diluted in Krebs solution to yield the indicated final drug concentrations in 0.1% DMSO.

### 2.1. Animals

The investigation conformed to the Guide for the Care and Use of Laboratory Animals (Institute for Laboratory Animal Research, National Academy of Sciences, 8th edition, 2011) and was approved by the Institutional Animal Care and Use Committee (ACORP 7435-1, VA Medical Center, Milwaukee, WI, USA). We used 12 and 12 eight-week-old male Brown Norway (BN) rats [32,33,34] for the IR experiments and for dose-response experiments, respectively.

### 2.2. Heart Isolation

The animals were anesthetized by the intraperitoneal injection of 100 mg/kg ketamine along with 1000 U heparin to prevent blood clotting. After a negative response to a noxious stimulus, the animals were euthanized by decapitation followed by thoracotomy. The aorta was cannulated distal to the aortic valve, and the heart was perfused retrograde with oxygenated Krebs solution (4 °C) containing (in mM) 148 Na^+^, 4.7 K^+^, 1.2 Mg^2+^, 1.6 Ca^2+^, 127 Cl^−^, 27.8 HCO_3_^−^, 1.2 H_2_PO_4_^−^, 1.2 SO_4_^2−^, 5.5 glucose, 2 pyruvate, 0.026 EDTA, and 5 U/l insulin. Both the venae cavae were ligated, and the heart was rapidly placed into a Langendorff support system and perfused at a constant pressure of 70 mmHg at 37 °C. The perfusate was equilibrated with ~95% O_2_ and ~5% CO_2_ to maintain a constant pH of 7.40 and filtered in-line (5 µm pore size). The isovolumetric left ventricular pressure (LVP) was measured with a saline-filled latex balloon (Radnoti LLC, Monrovia, CA, USA) inserted into the left ventricle. The diastolic LVP was initially adjusted to 10 mmHg at baseline (bl) so that any subsequent pressure increases reflected diastolic contracture. Systolic, diastolic, and developed (systolic–diastolic) LVP, and its maximal and minimal first derivatives (dLVP/dt_max_ and dLVP/dt_min_) were calculated as indices of ventricular contractility and relaxation, respectively. Electrodes attached to the right atrial and ventricular walls monitored atrial and ventricular electrocardiograms to calculate the spontaneous heart rate (HR) and identify arrhythmias. The rate-pressure product (RPP) as the product of developed pressure and HR was calculated to correct for HR-dependent changes in developed pressure. The coronary flow was measured in-line with an ultrasonic flowmeter (model T106X; Transonic Systems, Ithaca, NY, USA). All the analog signals were digitized (PowerLab/16 SP, AD Instruments; Castle Hill, Australia) and recorded at 200 Hz (Chart & Scope version 5.6.6, AD Instruments) for later analysis.

### 2.3. Protocols

**Figure 1:**
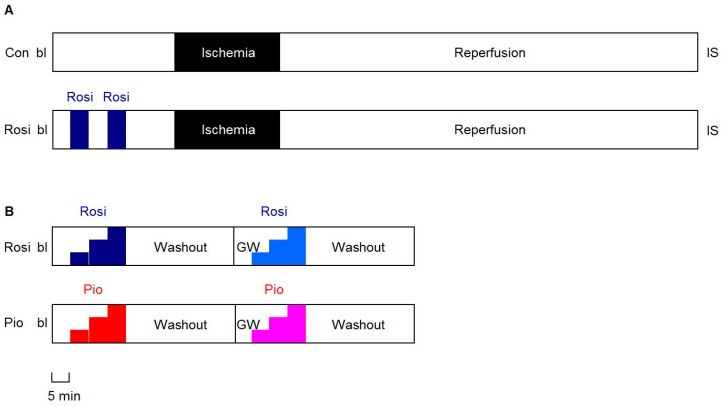
Shows the experimental protocols. The hearts were isolated from eight-week-old male Brown Norway rats and mounted in a Langendorff setup. After 20 min equilibration and a baseline (bl) reading, the hearts in the first set of experiments (n = 12; Panel (**A**)) were given rosiglitazone (Rosi, 50 µM) or a vehicle (Con) for two times, 5 min each with a 5 min washout period interspersed and followed by 15 min washout before 30 min of global no-flow ischemia and 120 min of reperfusion (IR) and subsequent infarct size (IS) determination. In a second set of experiments (Panel (**B**)), the hearts were given increasing concentrations (2, 10, and 50 µM) of either rosiglitazone (Rosi, blue colors; n = 6) or pioglitazone (Pio, red colors; n = 6) for 5 min each followed by 30 min washout. This series was repeated in the presence of 10 µM of the PPARγ antagonist GW9662 (GW, white). The hearts were not subject to IR or to IS determination.

The experimental protocols are illustrated in Figure 1. The baseline readings were taken after 20 min stabilization.

#### 2.3.1. IR Experiments

In this first set of experiments (n = 12; Figure 1, Panel A), rosiglitazone (Rosi, 50 µM) as a clinically used TZD was given as a preconditioning agent for two times, 5 min each with a 5 min washout period interspersed and followed by 15 min washout before 30 min of acute global no-flow ischemia and 120 min of reperfusion. This protocol was chosen to mimic our ischemic and other pharmacological preconditioning protocols [30,31,35,36] shown to be more effective than single exposure [31,37]. The control hearts (Con) received 0.1% DMSO as a vehicle only.

After removal of the hearts at the end of the experiments, the atria were discarded and the ventricles were cut into 2-mm transverse slices and incubated for 10 min in 1% 2,3,5-triphenyltetrazolium chloride in a 0.1 M KH_2_PO_4_ buffer (pH 7.4, 38°C) [38,39] which stains viable tissue red. The slices were digitally imaged on a green background, and the infarcted areas of each slice were measured automatically by planimetry using Image J 1.44i software (NIH, Bethesda, MD). The individual slice infarctions were weight-averaged to calculate the total ventricular infarct size (IS) per heart [32,40].

#### 2.3.2. Dose-Response Experiments

In a second set of experiments (n = 12; Figure 1, Panel B), the hearts were given increasing concentrations (2, 10 and 50 µM) of either rosiglitazone (Rosi) or pioglitazone (Pio) for 5 min each, without intervening IR or IS determination. After a 30 min washout, this series was repeated in the presence of the PPARγ antagonist GW9662 [12] at a concentration of 10 µM [11,41,42].

### 2.4. Fluorescence Measurement of Mitochondrial Redox State

Autofluorescence is widely used to measure mitochondrial electron transport in myocardial tissue [30,43,44]. Thus, the experiments were conducted in a light-blocking Faraday cage to assess the online autofluorescence of reduced NADH and oxidized FAD. The distal end of a trifurcated fiberoptic cable was placed gently against the left anterior ventricular wall while the proximal ends were connected to a modified spectrophotometer (Horiba, Piscataway, NJ, USA). At selected times, the shutter for excitation was opened for 2.5 sec intervals. The NADH fluorescence was excited at 350 nm followed by FAD fluorescence excitation at 488 nm. The NADH emissions were filtered at 460 ± 10 nm (Chroma Technology Corp., Brattleboro, VT, USA), FAD emissions at 540 ± 10 nm, and their respective fluorescence intensities were measured by photomultipliers.

### 2.5. Statistical Analysis

Unless otherwise indicated, all the values are expressed as a mean ± standard error of the mean (SEM) as %bl, and compared by analysis of variance (SigmaStat 3.5, Systat Software Inc., San Jose, CA, USA). If the F values were significant, Student-Newman-Keuls (SNK) post-hoc tests were conducted. Comparisons of only two groups were conducted with unpaired Student t-tests. All the results were considered statistically significant at *P* < 0.05 (2-tailed): *vs. Con (0 µM TZD).

## 3. Results

### 3.1. IR Experiments

The rosiglitazone (50 µM for 2 × 5 min) given before IR did not have a significant effect on myocardial function during its administration except for reversibly increasing coronary flow (Table 1). The infarct size was significantly increased in the rosiglitazone-treated hearts, but there was no significant positive or negative difference in the functional outcome after IR between the rosiglitazone-treated and control hearts.

### 3.2. Dose-Response Experiments

**Figure 2:**
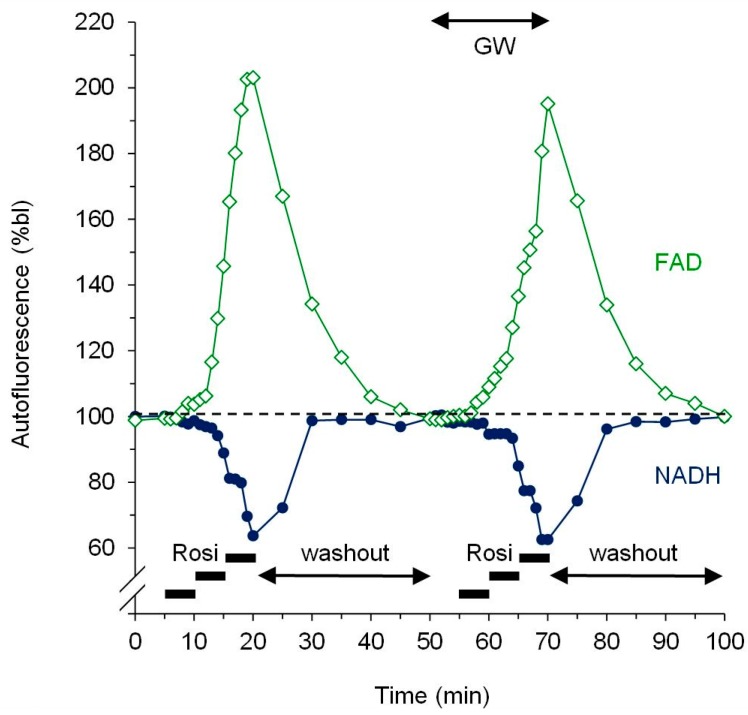
Shows representative time courses of NADH (closed blue circles) and FAD (open green diamonds) autofluorescence for an experiment with rosiglitazone (Rosi) in increasing doses from 0 to 50 µM (5 min each) first in the absence and then, after a 30 min washout period, in the presence of the PPARγ antagonist GW9662 (GW, 10 µM).

**Figure 3:**
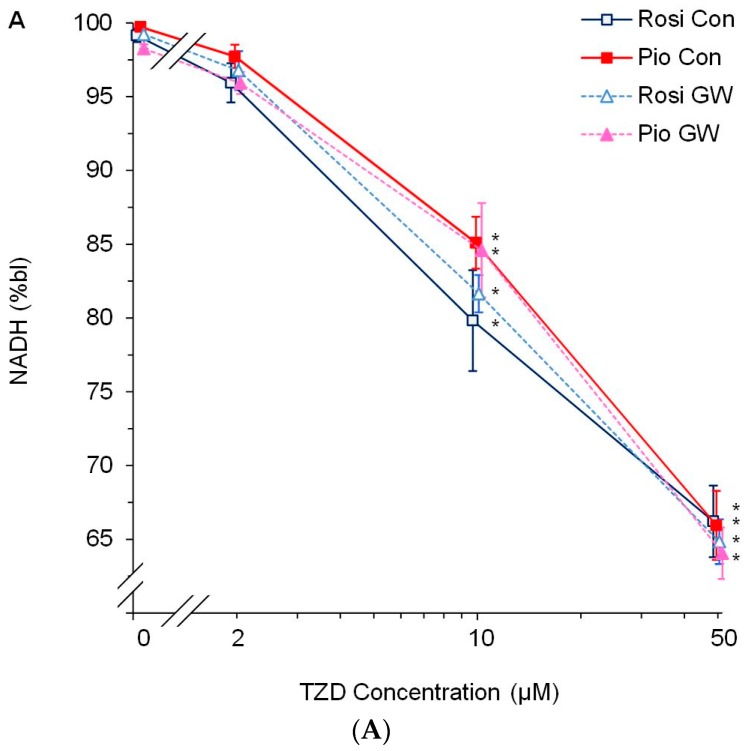

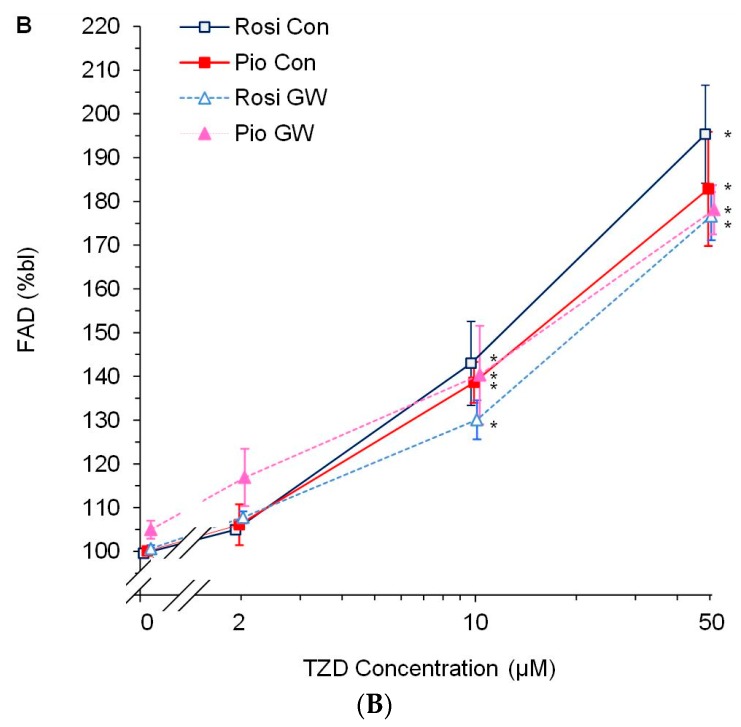
Shows a concentration-dependent decrease in autofluorescence of reduced NADH (Panel (**A**)) and a concomitant concentration-dependent increase in autofluorescence of oxidized FAD (Panel (**B**)) with administration of the thiazolidinediones (TZDs) rosiglitazone (Rosi, open dark-blue square) and pioglitazone (Pio, closed red square) compared to % baseline (bl). The PPARγ antagonist GW9662 (GW, 10 µM) did not alter the effect of rosiglitazone (open light-blue triangle) or pioglitazone (closed pink triangle) on NADH (Panel (**A**)) or FAD (Panel (**B**)) at any of the concentrations. Statistics: ANOVA followed by SNK post-hoc test with *P* < 0.05 (two-tailed) vs. * 0 µM TZD; n = 6 per group. Please note that the slight horizontal offset of curves for any given concentration is for visual purposes only.

Figure 2 shows the representative time courses of NADH and FAD fluorescence for an experiment with increasing doses of rosiglitazone (2, 10 and 50 µM) first in the absence and then, after a 30 min washout period, in the presence of the PPARγ antagonist GW9662 (10 µM). These dose-response experiments in the absence and presence of the PPARγ antagonist GW9662 revealed a PPARγ-independent increase in mitochondrial oxidation by rosiglitazone as evidenced by a dose-dependent decrease in NADH autofluorescence (Figure 2 and Figure 3A) and a dose-dependent increase in FAD autofluorescence (Figure 2 and Figure 3B). Neither of these was attenuated or abolished by GW9662 at a dose previously used to abolish endogenous and exogenous cardioprotection in the same [11] and in other models [41,42]. In order to test for a group- rather than a single drug-effect, the same dose-response curve was repeated with 2, 10, and 50 µM pioglitazone with essentially the same results (Figure 3).

## 4. Discussion

Our study has several key findings: In line with prior reports of cardiovascular side effects of TZDs [45,46], it confirms an increase in cardiac IR injury following the acute administration and washout of the TZD rosiglitazone in rat isolated hearts. A novel finding in this context however, is that in further dose-response experiments we found a considerable, rosiglitazone-induced, fully reversible increase in mitochondrial oxidation as assessed by decreased NADH and increased FAD autofluorescence. This finding was independent of PPARγ activation as it was not abolished by the PPARγ antagonist GW9662. Moreover, all the observed rosiglitazone effects on the mitochondrial redox state were replicated with another member of the TZD family, pioglitazone, which suggests a group- rather than a mere single drug-dependent side effect on mitochondrial function.

### 4.1. PPARγ Activation and Myocardial Protection: Friend or Foe?

Several options are available to investigate the specific aspects of a particular signaling pathway. For example, the expression, modification and/or activity of a certain protein/enzyme can be measured. An agonist can be used to activate a certain pathway. Or a specific antagonist can be used to attenuate or abolish a certain finding. Using the latter, we have previously shown in a consomic rat model of resistance against myocardial IR that the specific PPARγ antagonist GW9662 prevented endogenous and exogenous cardioprotection [11]. Thus, PPARγ activation is a critical part of cardioprotective pathways that can be initiated by different triggers upstream of PPARγ [12].

Conversely, administration of specific PPARγ agonists should be able to mimic the above phenotype and activate a cardioprotective pathway directly without the need for, e.g., ischemic or anesthetic preconditioning, and their acutely cardio-depressant and other side effects [12]. While numerous experiments with the PPARγ antagonist GW9662 have largely shown myocardial protection against IR injury by PPARγ activation [2,12,47,48,49,50,51], experiments with agonists like TZDs have revealed less clear results, particularly under pathological conditions [52]. To the contrary, but in line with our findings, the TZD-triggered aggravation of myocardial outcome was shown in animal studies [13,14,15,16,17] and in humans [18,19]. The apparently contradictory findings among some of these studies may be due to differences in the species and experimental models being used, the protocol and duration of TZD administration, co-morbidities, and/or related to the used dosage.

### 4.2. Specificity of TZDs for PPARγ Activation

Conclusions from experiments using agonists and/or antagonists generally rely on their respective specificity for any given pathway or receptor. Thus, it is possible that TZDs have PPARγ-independent binding sites [53] that can influence cardiovascular outcome directly or indirectly [54,55,56]. Reports about the deleterious effects of TZDs without their prevention by the use of a specific PPARγ-antagonist [13,15,16,17] may, therefore, be due to PPARγ-independent side effects as previously discussed by Feinstein and colleagues [54]. Moreover, these reports pose the question of whether those side effects are specific to individual members of the TZD group or side effects of the TZD group as a whole because of their chemical similarities [53]. The use of more than one member of the TZD group within the same study and demonstration of failure to abolish the observed effect with GW9662 can add clarification in these regards.

### 4.3. TZDs Affect Mitochondrial Function

Our knowledge about the cardiac mitochondrial side effects of TZDs is sketchy [54]. An in-vivo and in-vitro study in mice [57] reported rosiglitazone to cause dysfunction of cardiac mitochondria as evidenced by decreased mitochondrial respiration and substrate oxidation, as well as decreased complex I and IV activities. Rosiglitazone also increased superoxide production from complexes I and III. Neither genetic PPARγ deletion nor the PPARγ antagonist GW9662 prevented these effects. Moreover, these findings were associated with decreased ATP synthesis and increased cardiac dysfunction during rosiglitazone administration. The authors emphasize that, similarly to our findings, these were dose-dependent effects found at concentrations of 10 µM and higher.

Reactive oxygen species (ROS) are produced from different intracellular sources with mitochondria and different mitochondrial electron transport chain complexes being the major sources in cardiomyocytes [58]. While inhibition of the mitochondrial electron transport chain at specific sites of complex I and/or III [58] can lead to oxidative stress, the latter is not necessarily proof of a blockade but could also be caused by an increase in electron transport, as is the case with uncoupling [59]. Indeed, mitochondrial uncoupling makes oxidative phosphorylation less efficient, increases ROS, and—if not countered by increased delivery of the reducing equivalents, NADH and FADH_2_, through the Krebs cycle—can lead to a decreased membrane potential and ATP synthesis. Thus, a decrease in membrane potential or ATP synthesis or an increase in ROS production cannot distinguish between the uncoupling and blockade of mitochondrial electron transport, unless the mitochondrial redox state is measured. In this context, our results suggest a dose-dependent and reversible mitochondrial oxidation by two different TZDs independent of PPARγ activation. Excessive mitochondrial oxidation can lead to decreased ATP synthesis and excessive ROS production, all of which can damage the myocardium and/or lead to increased sensitivity to subsequent IR.

### 4.4. Study Limitations and Summary

This study needs to be interpreted within its natural constraints. We used one species and an acute rather than chronic experimental IR injury model. Mitochondrial function was assessed by two different redox state measurements in intact hearts, but not by mitochondrial oxygen consumption in isolated mitochondria, and we did not assess ROS production, membrane potential or ATP synthesis, neither of which would have added to differentiating severe blockade of mitochondrial electron transport from uncoupling. The IR injury assessment was limited to one TZD and one dose given twice acutely and washed out before IR. On the other hand, rosiglitazone’s effects on the redox state, with or without the PPARγ antagonist GW9662, were closely mimicked by pioglitazone; the NADH results mirrored the FAD measurements; and the GW9662 results were nearly identical to the ones in its absence, serving as internal controls. Although we have not conducted a formal dose-response study with the PPARγ antagonist GW9662 or Western blot analysis, we have chosen a dose (10 µM) commonly used to block PPARγ in the isolated heart [11] and isolated cardiomyocyte studies [41,42]. Demonstration of a change in infarct size remains the gold standard for in- and ex-vivo studies on cardioprotective or -toxic agents and strategies, even in the absence of significant functional changes. Within limits, compensatory mechanisms, such as e.g., mildly increased intracellular calcium in surviving cardiomyocytes, can make up for the loss of function in the infarcted myocardium. The infarct size is among the more sensitive parameters with earlier responses to IR than functional changes [36]. We exposed hearts from non-diabetic animals to an acute dose of rosiglitazone; chronic exposure in diabetic individuals would require the study of chronically diabetic and, thus, hyperglycemic hearts in the control group vs. normoglycemic hearts chronically exposed to a TZD which would complement but not replace the findings in the present study and add additional confounders to the study.

In summary, our study in rat isolated hearts suggests that the off-target effects of the TZDs, rosiglitazone and pioglitazone, include a significant degree of mitochondrial oxidation associated with aggravated myocardial IR injury that can help explain the reported increase in adverse cardiac events. Because of the large number of diabetic patients worldwide who are chronically treated with TZDs, it is important to unravel the mechanisms of these adverse effects and their clinical consequence in future studies, including diabetic IR models, in order to improve overall patient outcome while minimizing unwanted side effects.

## Figures and Tables

**Table 1:** Myocardial Function and Infarct Size. This table shows myocardial function during the application of the thiazolidinedione rosiglitazone (Rosi, 50 µM, n = 5), and myocardial function and infarct size at 120 min reperfusion following 30 min global no-flow ischemia compared to control (Con, n = 7) rat isolated hearts.

	During Application		120 min Reperfusion	
	Con	Rosi	Con	Rosi
sysLVP (%bl)	95.5 ± 2.0	101.9 ± 2.8	70.5 ± 4.9	60.3 ± 3.9
diaLVP (mmHg)	10.5 ± 2.1	9.9 ± 0.8	32.3 ± 4.3	31.8 ± 2.7
devLVP (%bl)	92.6 ± 3.8	102.4 ± 3.6	33.8 ± 3.7	29.7 ± 1.0
RPP (%bl)	96.4 ± 5.5	101.3 ± 4.4	33.5 ± 3.7	30.9 ± 0.7
dLVP/dt_max_ (%bl)	94.4 ± 3.5	106.6 ± 4.7	35.9 ± 2.6	32.8 ± 1.3
dLVP/dt_min_ (%bl)	97.2 ± 2.4	100.6 ± 4.5	38.9 ± 5.0	30.4 ± 1.1
HR (%bl)	105.3 ± 2.8	98.8 ± 2.2	98.4 ± 2.7	104.3 ± 1.6
CF (%bl)	100.0 ± 1.0	* 124.0 ± 6.1	64.6 ± 5.3	64.5 ± 3.0
IS (%)			36.2 ± 3.4	* 45.3 ± 0.7

bl = baseline; LVP = left ventricular pressure; sys = systolic; dia = diastolic; dev = developed; RPP = rate-pressure product; dLVP/dt_max_ = contractility; dLVP/dt_min_ = relaxation; HR = heart rate; CF = coronary flow; IS = ventricular infarct size. All values are mean ± standard error of the mean of %bl unless otherwise indicated. Statistics: unpaired student *t*-test with * *P* < 0.05 (two-tailed).
